# Long-term outcome following surgical treatment of posttraumatic tethered cord syndrome: a retrospective population-based cohort study

**DOI:** 10.1038/s41393-022-00752-7

**Published:** 2022-01-19

**Authors:** Vasilios Stenimahitis, Alexander Fletcher-Sandersjöö, Charles Tatter, Adrian Elmi-Terander, Erik Edström

**Affiliations:** 1 Department of Neurology, Karolinska University Hospital, Solna, Sweden; 2 Department of Clinical Neuroscience, Karolinska Institutet, Solna, Sweden; 3 Department of Neurosurgery, Karolinska University Hospital, Solna, Sweden

**Keywords:** Outcomes research, Spinal cord diseases

## Abstract

**Study design:**

Retrospective population-based cohort study.

**Objective:**

To investigate the long-term outcome following surgery for posttraumatic spinal cord tethering (PSCT).

**Setting:**

Publicly funded tertiary care center.

**Methods:**

Patients surgically treated for PSCT between 2005–2020 were identified and included. No patients were excluded or lost to follow-up. Medical records and imaging data were retrospectively reviewed.

**Results:**

Seventeen patients were included. Median age was 52 (23–69) years and 7 (41%) were female. PSCT was diagnosed at a median of 5.0 (0.6–27) years after the initial trauma. Motor deficit was the most common neurological manifestation (71%), followed by sensory deficit (53%), spasticity (53%), pain (41%) and gait disturbance (24%). Median follow-up time was 5.1 (0.7–13) years. Fifteen patients (88%) showed satisfactory results following untethering, defined as improvement or halted progression of one or more of the presenting symptoms. Treatment goals were met for motor symptoms in 92%, sensory loss in 100%, spasticity in 100%, gait disturbance in 100% and pain in 86%. Statistically, a significant improvement in motor deficit (*p* = 0.031) and syrinx decrease (*p* = 0.004) was also seen. A postoperative complication occurred in four patients: three cases of cerebrospinal fluid leakage and one postoperative hematoma. Two patients showed a negative surgical outcome: 1 with increased neck pain and 1 with left arm weakness following the postoperative hematoma.

**Conclusion:**

Surgical treatment of PSCT results in improved neurological function or halted neurological deterioration in the vast majority of patients.

## Introduction

**Fig. 1:**
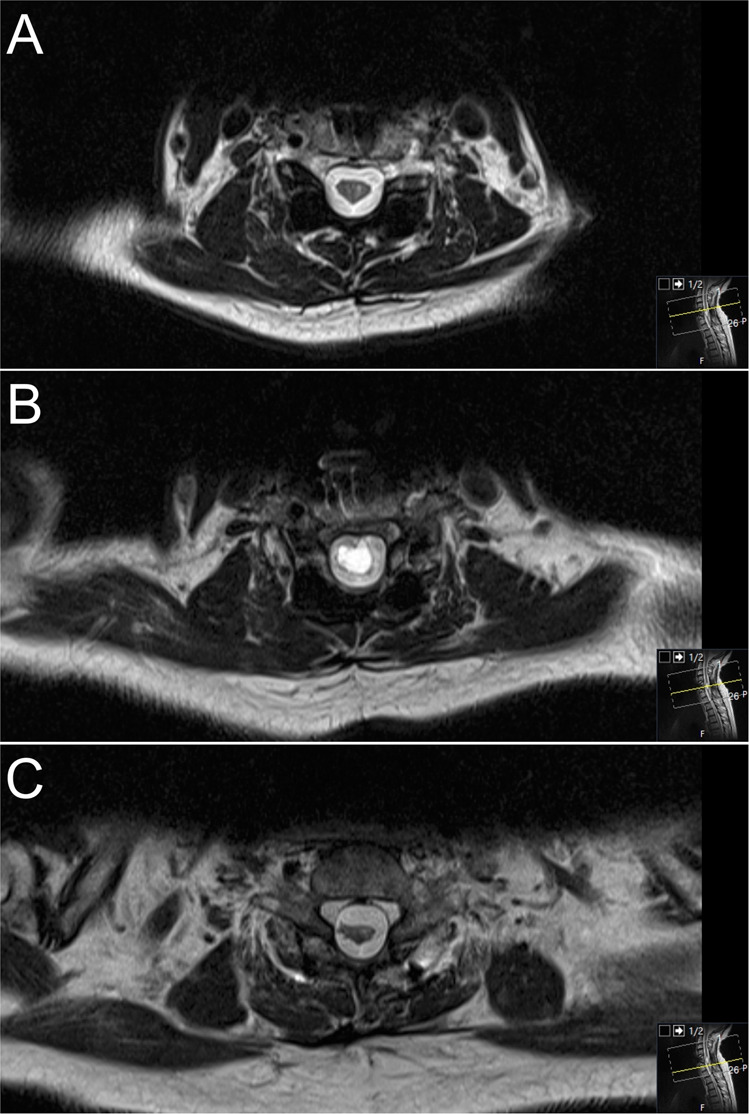
MRI of posttraumatic tethered cord. Axial T2 magnetic resonance images showing a posttraumatic tethered cord with syrinx formation above the cyst (**A**), in level with the cyst (**B**) and below the cyst (**C**).

**Fig. 2:**
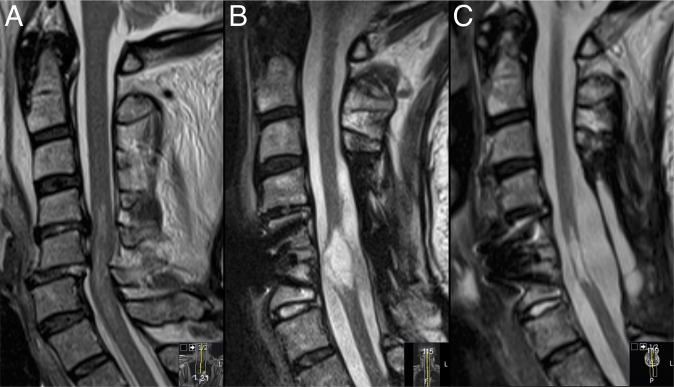
Time points in a  case of PSCT. Sagittal T2 magnetic resonance images immediately after trauma (**A**), tethering and syringomyelia formation 3 years after trauma (**B**), and following surgical untethering (**C**).

Spinal trauma may lead to spinal cord injury and different degrees of associated neurological deficits at and below the level of the lesion [1]. Delayed syrinx or cyst formation in combination with a progressive neurological decline has been generally recognized and accepted as a result of traumatic spinal cord injury [2, 3]. Posttraumatic spinal cord tethering (PSCT) (Figs. 1, 2) including excessive arachnoid scar formation is recognized as an underlying factor for cyst formation or posttraumatic syringomyelia (PS) [4–8]. Due to the fact that not all clinical aggravations in chronic spinal cord injuries are accompanied by PS, the term progressive posttraumatic myelopathy (PPM) was introduced to emphasize the fact that neurological decline as a result of tethering may occur with or without a co-existing PS formation [3, 4]. As a result of PPM, spinal cord injury patients may develop a progressive ascending neurological deterioration within a time frame that ranges from a few months up to several decades after the initial trauma [1, 3, 6, 7, 9–12]. It has been suggested that 0.3–4.5% of patients with spinal cord injury develop clinically significant PPM [1, 3]. A higher incidence, up to 30%, is suggested by radiological and autopsy studies [9]. The symptomatology of PPM consists of impaired motor and sensory function, abnormality of temperature sensation, worsening of spasticity, pain, autonomic impairment, hyperhidrosis, Horner’s syndrome, and bowel and bladder dysfunction [1, 3, 5–15].

The pathophysiology of PPM is not fully understood. The occurrence of PS is mainly correlated to the degree of arachnoid adhesions and obstruction of cerebrospinal fluid circulation [1, 5, 13]. Traction of the tethered cord, resulting in mechanical distention and subsequent local ischemia has also been suggested as a causal factor [7]. The treatment is surgical, and the main objective is to prevent further neurological decline [1]. Surgical approaches include arachnoidolysis, duraplasty, and fenestration or shunting of PS [1, 10, 11, 13].

From a neurosurgical perspective, untethering of the spinal cord is the goal of treatment and for the purpose of this text, the term PSCT is a better match than PPM. Hence, PSCT will be taken to include both concepts. Similarly, we will not try to delineate between posttraumatic syringomyelia and cyst formation, and in the context of PSCT view them as part of the same continuum. The term posttraumatic syringomyelia (PS) will be used.

Uncertainty exists regarding the management of PS and a recent meta-analysis identified that the past four decades have failed to produce a consensus regarding its treatment [16]. Similarly, a consensus panel agreed that the evidence was poor but suggested detethering with dural expansion as a first treatment strategy for PSCT and PS [9].

In this study, we present our institutional experience from surgical treatment of PSCT, with special emphasis on the management and results in relation to concomitant PS. We aim to describe the clinical characteristics of patients treated for PSCT with or without concomitant PS, to present the surgical approach and methodically examine the long-term outcomes following surgical treatment.

## Methods

### Patient selection

In the Stockholm region, patients who have suffered a traumatic spinal cord injury are offered coordinated inpatient and outpatient rehabilitation in accordance with a structured health care program. After discharge from the inpatient rehabilitation unit, the patients are admitted to outpatient rehabilitation at the “Spinalis” spinal cord injury outpatient clinic. Spinalis provides lifelong follow-up for patients with spinal cord injuries with a dedicated team of health care professionals that when appropriate initiate additional investigations, including MRI and referrals to the neurosurgical department for evaluation. All referrals for PSCT are evaluated at the Department of Neurosurgery, Karolinska University Hospital. This is the only neurosurgical center in the Stockholm region and a publicly funded and owned tertiary care center serving a region of approximately 2 million inhabitants. Thus, there was no selection bias in this study. Surgical decisions were made in consensus by three senior consultant neurosurgeons following a multidisciplinary conference attended also by neurologists, neuroradiologists and rehabilitation specialists. All SCI patients, where deterioration could be attributed to tethering, were discussed at these conferences with the intention to treat all that could benefit from surgery.

All adult patients (≥18 years) surgically treated for PSCT at the study center, between 2005 and 2020, were included in the study. Patients were identified through the hospital’s surgical management software Orbit (Evry Healthcare Systems, Solna, Sweden). No patients were excluded. Medical records and imaging data from digital hospital charts were retrospectively reviewed using the health record software TakeCare (CompuGroup Medical Sweden AB, Farsta, Sweden). The outcome was assessed by change in PS size and clinical status. The study was approved by the Swedish Ethical Review Authority (Dnr: 2020–02086) who waived the need for informed consent.

### Surgical technique and follow-up

Following referral, a detailed neurological examination was performed on all patients. Special attention was given to any differential diagnoses that might explain the patients’ symptoms. Patients were offered surgery if they had an MRI-verified spinal cord injury and symptoms that could be attributed to PSCT. Prior to surgery, the spinous process of the vertebra above the lesion (if thoracic or lumbar) was identified using CT guidance and marked with injection of a sterile carbon suspension. For cervical lesions, the head was fixed in a Mayfield clamp and fluoroscopy was used to identify the correct level. With the patient in the prone position, a posterior midline approach was used. Laminectomy was performed in most cases, using an ultrasonic bone scalpel (Misonix Inc., Farmingdale, New York, USA). Intraoperative neurophysiological monitoring was applied in three cases. Ultrasound registration was performed before opening of the dura. Under the microscope, the dura was incised in the midline and held open by sutures. The arachnoid adherences were dissected sharply, and the tethered area was exposed. Untethering was primarily performed of the dorsal and lateral aspects of the spinal cord to the level of the nerve root foramina. Subsequent anterior untethering was performed only when deemed possible. Efforts were made to restore CSF passage. The size of any present PS was assessed both visually and by ultrasound. If untethering did not result in at least 50% decrease in size of the PS, a syringo-subarachnoid shunt or fenestration was considered, based on the surgeon’s preference. For every case, expansion duraplasty was performed with a 1.5 cm wide graft using Lyoplant (B.Braun, Melsungen, Germany), with intended watertight dura closure using non-resorbable sutures. Microplates (CMF Medicon Surgical Inc., Jacksonville, Florida) were placed laterally on adjacent lamina to serve as anchoring points and sutures were used to tent the dura and lift it from the cord. Fibrin sealant (Evicel, Ethicon, Somerville, NJ, USA) was applied to the suture line. The soft tissue layers were then sutured individually to close the wound.

Following surgery, patients were kept on bedrest for at least 24 h and thereafter mobilized. All patients were discharged to a rehabilitation facility before returning home. In adherence with routine protocols, all patients underwent a follow-up MRI (Fig. 2) and clinical examination by the treating surgeon after 3 months. In addition, all patients were followed, at least annually, by physiotherapists and rehabilitations specialists at the Spinalis Clinic. As the same electronic records are used in the entire region, long-term follow-up data (including available data on ASIA, EQ5D, FIM, and Braden) were included in this study. The outcome of “improved”, “unchanged”, or “worsened” was based on the longest follow-up available for each patient. Since the aim of surgery was to prevent and arrest further neurological deterioration, the definition for reaching the surgical goals was subjective improvement of preoperative symptoms or unchanged postoperative status.

### Quality of life measures

Spinal cord injuries are graded according to the American Spinal Injury Association (ASIA) grading scale, which assesses sensory and motor functions to describe the severity of the injury. The more severe the injury, the less likely a recovery will occur. The scale ranges from A to E, where A represents a complete spinal cord injury and E represents normal function [17].

The EQ-5D-3L measures Health Related Quality of Life (HRQoL) and consists of two parts. The first part is a descriptive system in which the respondents classify their health in 5 dimensions with three severity levels [18]. The responses can be expressed as a single value, the EQ-5D_index_, where 0 represents dead and one represents full health. Importantly, some health states are on a group level considered worse than death and are assigned negative values [19]. The United Kingdom value set was used to calculate the EQ-5D_index_ [20]. The second part is the EQ visual analogue scale (EQ_VAS_), where the respondents rate their current health between 0 (worst imaginable health) and 100 (best imaginable health).

The Functional Independence Measure (FIM) is an 18 items instrument, designed to measure disability irrespective of underlying diagnosis and includes aspects of dependence and self-care in relation to everyday activities such as eating, dressing, toileting, transfers, and mobility. It also covers aspects of cognitive function such as comprehension, social interaction and memory. Tasks are rated on a 7-point scale (range from total assistance to complete independence). Scores range from 18 (lowest) to 126 (highest). They are generally rated at admission and upon discharge [21–23].

Braden scale is a tool used to predict the risk of developing pressure ulcers. Braden scale includes six categories: sensory perception, moisture, activity, mobility, nutrition and friction & shear). Each category is rated on a 1–4 scale (the category friction & shear is rated on a 1–3 scale), combining into a total possible score of 23. A higher score means a lower risk of developing a pressure ulcer (a score of 23 means that there is no risk for developing a pressure ulcer and a score of 6 means the most severe risk of developing a pressure ulcer) [24].

### Statistics

Shapiro–Wilks test was used to evaluate the normality of the data. As all continuous data significantly deviated from a normal distribution pattern (Shapiro–Wilks test *p* value < 0.05), it is presented as median (range) and categorical data as numbers (proportion). Continuous quality of life data was presented as mean ± standard deviation as well to illustrate significant differences. McNemar’s test (paired categorical data) was used to compare neurological symptoms and syrinx status before and after surgery. Analyses were conducted using the statistical software R. Statistical significance was set at *p* < 0.05.

## Results

### Baseline characteristics

During the study period, 17 patients with PSCT were diagnosed and surgically treated. No patients were excluded or lost to follow-up. Seven (41%) of the patients were female, and the median age was 52 years (range 23–69). Traffic accidents were the most common type of trauma associated with later tethering (*n* = 7, 41%), followed by falls (*n* = 5), penetrating trauma (*n* = 3) and diving accidents (*n* = 2). The neurological deterioration and subsequent diagnosis of PSCT was made at a median of 5.0 years (range 0.6–27) after the initial trauma, and the most common manifestation of cord tethering was motor deficit (*n* = 12, 71%), followed by sensory deficit (*n* = 9, 53%), spasticity (*n* = 9, 53%), pain (axial or radicular) (*n* = 7, 41%) and gait disturbance (*n* = 4, 24%). At the time of tethering diagnosis, eight patients (47%) were ASIA grade A, i.e., a complete lack of motor and sensory function below the level of injury. Based on the preoperative magnetic resonance images (MRI), the most common tethering location was the cervical spine (*n* = 8, 47%), 12 patients (71%) had an associated PS, and 9 (53%) showed increased intramedullary signal intensity (Table 1).

**Table 1:** Baseline characteristics.

Variable	Value (*n* = 17)
Female sex	7 (41%)
Age (years)	52 (23–69)
Surgical treatment for initial trauma	12 (71%)
Time from trauma to diagnosis of tethered cord (years)	5.0 (0.6–27)
Trauma mechanism	
Traffic accident	7 (41%)
Fall	5 (29%)
Penetrating trauma	3 (18%)
Diving accident	2 (12%)
Radiology	
Level of tethering	
Cervical	8 (47%)
Cervicothoracic	5 (29%)
Thoracic	2 (12%)
Thoracolumbar	2 (12%)
Lumbar	0 (0%)
Syrinx	12 (71%)
Increased intramedullary signal intensity	9 (53%)
Manifestation of tethering	
Pain	7 (41%)
Motor deficit	12 (71%)
Sensory deficit	9 (53%)
Spasticity	9 (53%)
Gait disturbance	4 (24%)
ASIA impairment scale	
A	8 (47%)
B	3 (18%)
C	0 (0%)
D	6 (35%)
E	0 (0%)

Values are expressed as median (range) or numbers (proportion).

### Treatment

The median time between diagnosis of the tethered cord and untethering surgery was 8.2 months (range 2.2–136). All patients were treated with laminectomy, untethering, expansion duraplasty and tenting. A syringo-subarachnoid shunt was inserted in 2 cases and fenestration without shunting performed in 1 case. Four postoperative complications occurred. This included three cases of cerebrospinal fluid (CSF)-leak with pseudomeningocele, of which one required surgical repair while the other two were managed with a lumbar drain. In addition, one patient suffered from a postoperative hematoma that required emergency surgery. No patients died during follow-up (Table 2).

**Table 2:** Outcome data.

Variable	Value (*n* = 17)
Time from diagnosis of tethering to surgery (months)	8.2 (2.2–136)
Intraoperative complication	0 (0%)
Follow-up time (years)	5.1 (0.7–13)
Posttraumatic syringomyelia change (*n* = 12)	
Complete resolution	3/12 (25%)
Partial resolution	6/12 (50%)
Unchanged	2/12 (17%)
Unknown	1/12 (8%)
Postoperative complication	4 (24%)
Ibanez grade 2a	2
CSF-leak treated with lumbar drain and antibiotics	2
Ibanez grade 2b	2
CSF-leak requiring surgical revision	1
Postoperative hematoma requiring surgical revision	1
Death during follow-up	0 (0%)

Values are expressed as median (range) or numbers (proportion). Abbreviations: *CSF* Cerebrospinal fluid.

### Outcome

The median follow-up time was 5.1 years (range 0.7–13). In total, 15 patients (88%) showed satisfactory results following untethering surgery, defined as improvement or halted progression of one or more of the presenting signs or symptoms. More specifically, surgical untethering showed satisfactory results for motor symptoms in 92% (11 of 12), sensory loss in 100% (9 of 9), spasticity in 100% (9 of 9), gait disturbance in 100% (4 of 4) and pain in 86% (6 of 7). In addition, paired testing showed that there was also a significant improvement in motor deficit (*p* = 0.031) and PS status (*p* = 0.004) following surgery. A more detailed account of the functional outcomes can be seen in Table 3.

**Table 3:** Preoperative symptoms and postoperative improvement.

Symptom	Preoperative incidence (*n*)	Satisfactory outcome		Negative outcome	*P* value (paired testing)
		Improved	Unchanged/halted progression	Aggravated	
Pain	7	1 (14%)	5 (71%)	1 (14%)	1.000
Motor deficit	12	5 (29%)	6 (36%)	1 (8%)	**0.031**
Sensory deficit	9	4 (44%)	5 (56%)	0 (0%)	0.125
Spasticity	9	2 (22%)	7 (78%)	0 (0%)	0.500
Gait disturbance	4	3 (75%)	1 (25%)	0 (0%)	0.250
PS	12	9 (75%)	2 (17%)	0 (0%)	**0.004**

Values are expressed as numbers (proportion). Bold text indicates a statistically significant correlation (*p* < 0.05). Abbreviations: *PS* Posttraumatic syringomyelia.

Two patients had a negative surgical outcome. One of these was a patient with satisfactory radiological results but unchanged motor function and increased neck pain. The other was the patient who developed a postoperative hematoma requiring emergency surgery, resulting in increased left arm weakness (Table 3). All patients with preoperative AISA D (*n* = 6) reached the treatment goal of improved or halted progression. No patient worsened. Despite these improvements, all patients were still classified as AISA D after surgery. In fact, there were no patients who changed their ASIA score after surgery.

The EQ5D_index_ for this group of patients was 0.224 ± 0.387 (*n* = 9, 8 missing). While EQ_vas_ was 47 ± 18. Two patients had negative EQ5D_index_ values indicating a HRQoL worse than death. FIM was available for 15 patients (2 missing). During the study period, FIM motor score was increased on a group level from 43 ± 27 to 65 ± 26 (*p* = 0.008). Eleven patients improved, two were unchanged and two worsened. All patients had the maximum of 35 points on the cognitive scoring part. Data regarding Braden scale was available for 13 patients (range 11–23, 4 missing). Seven patients had ≥19 and 6 had ≤18, indicating that 46% had a moderate risk for developing a pressure ulcer.

Of the 12 patients with PS, 25% showed complete resolution, 50% partial resolution, and the rest remained unchanged (Table 2). Nine of these patients were surgically treated with untethering and expansion duraplasty with satisfactory decrease in PS size. The remaining three underwent an additional PS specific treatment, with fenestration performed in one patient, resulting in complete resolution, while a syringo-subarachnoid shunt was placed in the other two cases resulting in partial decrease of PS size for both.

## Discussion

This study aimed to analyze the long-term outcome after surgical treatment of PSCT. Our key conclusions are: (a) the most common clinical sign leading to surgery was motor deficit (followed by sensory deficit and spasticity), (b) neurological improvement or discontinuation of deterioration of one (or more) of the presenting symptoms was reported in a majority of patients (88%), (c) an associated PS was identified in 12 (71%) patients and typically resolved after surgery, (d) a limited number of postoperative complications were reported.

The surgical approach with untethering of the cord, expansion duraplasty with tenting of the dura and, when necessary, PS drainage aims to prevent the progressive neurological decline associated with PSCT [2]. A favorable outcome was considered one associated with improvement or unaffected neurological status following surgery [1, 2, 7, 15]. The surgical approach with duraplasty and tenting of the dura seems to be important for good long-term outcome. In our material, one patient underwent untethering without duraplasty and tenting. This patient had gradual recurrence of symptoms within 6 months. A repeat surgery, with duraplasty and tenting, was performed 4 years later and the tethering symptoms resolved.

Motor deficit was the most common neurological manifestation of PSCT in our series (71%). The decline of motor function (loss of strength) as a common presenting sign is also reported in other studies [2–4, 7, 14]. A comparison of preoperative symptoms with the postoperative neurological status in this subset showed satisfactory outcome in almost all patients (11 of 12) with five patients presenting with improvement of motor deficit and six patients showing halted progression of further neurological deterioration. This is supported by previous studies describing surgical results regarding functional status in patients with PSCT and PS where 89% of patients reported arrest of progressive loss of sensory and/or motor function [2].

The neurological evaluation in our patient group, at follow-up 5.1 years post-surgery (range 0.7–13), showed favorable results with zero negative outcome regarding other clinical parameters that were co-indicators for surgery such as: (a) sensory deficit (improvement 44% and halted progression in 56%), (b) spasticity (improvement in 22% and halted progression in 78%) and (c) gait disturbance (improvement 75% and halted progression 25%). In the subset of patients presenting with pain in the preoperative evaluation (7/17), one reported progress of pain post-surgery while the other six patients showed improved status or halted progression of pain related to progressive PSCT. Published data on surgical outcome in patients with PSCT and PS, based on patient-focused outcome questionnaires, showed that 26% of patients that present with progressively worsening neuropathic pain reported a negative outcome post-surgery with deterioration of neuropathic pain, whereas 74% reported either decreased neuropathic pain or unchanged status [2]. Other studies analyzing outcome after surgical treatment of PS and PSCT, according to questionnaire results, show that upon evaluation 1 year after surgery, substantial improvement in neuropathic pain was reported in 55.6% of the patients [4]. Thus, we found that the surgical treatment strategy for our patients produced results that were equal or superior to those previously published regarding improved neurological function. Our one case of increased pain despite a favorable radiological outcome was post-laminectomy pain while the case of neurological decline was due to a postoperative hematoma.

The postoperative complications related to untethering of the cord with scar removal (with or without PS drainage) are overall uncommon and well tolerated by the patients with the vast majority of the patients (88%) showing satisfactory outcome [1]. In our study, four postoperative complications were reported, including three cases of cerebrospinal fluid leakage and one case of postoperative hematoma requiring acute surgery. In another large series of 362 patients, postoperative complication with CSF leakage was reported in 4% [1, 2].

Intraoperative neurophysiological monitoring (IONM) was applied in only three cases. Although none of the complications above could have been avoided by using IONM, our experience with related surgeries for intramedullary tumors shows a great value of IONM to increase surgical safety [25]. IONM, using somatosensory evoked potentials (SEP), motor-evoked potentials (MEP) and D-wave have been shown to decrease the incidence of postoperative functional neurological decline in intramedullary tumor surgery [26–28]. The sporadic use in this series reflects a historic situation with limited access to IONM. The benefits of INOM in untethering surgery, has previously been emphasized [29]. We suggest that IONM should be used in all cases of surgical untethering.

Neurological decline due to PSCT may be accompanied by the development of PS [1, 2, 14]. The development of PS is related to the extent of the arachnoid obstruction and adhesion [13, 30]. In 71% of the patients in our study (12/17), PS development occurred. In two of these cases a syringo-subarachnoid shunt was introduced and in one case a fenestration was performed. In the larger series with 362 patients [2], placement of a shunt tube in addition to untethering and duraplasty was performed in 20% of the cases. As our series only contained two cases of shunt placement, we could not statistically evaluate its impact on outcome.

The available data regarding quality of life measurements (EQ5D, FIM, and Braden), indicate, not surprisingly, a lower quality of life compared to previously published data on the Swedish general population The EQ5D_index_ and EQ_vas_ were 0.224 ± 0.387 and 47 ± 18, respectively. These numbers can be compared to those of a sample of the general population in Sweden, where the EQ5D_index_ and EQ_vas_ where 0.75 ± 0.25 and 72 ± 22, respectively, indicating a much worse quality of life for the study group [31]. The EQ5D_index_ was below zero in two cases, indicating a very poor quality of life. Half of the patients are at risk for pressure ulcers according to the Braden scale. The data on quality of life were not systematically acquired at specific time points but is rather a reflection of changes during the follow-up period. We noticed a significant improvement regarding motor scores in FIM, however, this finding should be interpreted with caution since it represents the sum of rehabilitation measures and cannot be solely attributed to the surgery.

## Limitations

The data collection and the review of the digital hospital charts were performed retrospectively. The small sample size limited statistical analyses. Nonetheless, our findings underline the satisfactory results following untethering surgery and highlight duraplasty and arachnoidolysis as a treatment approach in order to prevent further neurological deterioration from PS formation and progressive posttraumatic myelopathy.

## Conclusions

Surgical treatment of PSCT resulted in improved neurological function or halted neurological deterioration in the vast majority of patients.

## Data Availability

The datasets generated for this study are available on request to the corresponding author.
